# Enhanced brain myelination and cognitive development in young children associated with milk fat globule membrane (MFGM) intake: a temporal cohort study

**DOI:** 10.1007/s00429-025-02907-7

**Published:** 2025-04-19

**Authors:** Sean C. L. Deoni, Jennifer Beauchemin, Viren D’Sa, RESONANCE Consortium

**Affiliations:** 1 Advanced Baby Imaging Lab, Warren Alpert Medical School at Brown University, Providence, RI 02912 USA; 2 Department of Pediatrics, Warren Alpert Medical School at Brown University, Providence, RI USA

**Keywords:** Infant nutrition, Infant brain development, Myelination, Neurodevelopment, Cognitive development

## Abstract

Myelination is an important neurodevelopmental process that facilitates efficient brain messaging and connectivity, and contributes to the emergence and refinement of cognitive skills and abilities. Healthy maturation of the myelinated white matter requires coordinated delivery of key nutritional building blocks, including short and long-chain polyunsaturated fatty acids, phospholipids, and sphingolipids. While many of these nutrients are amply supplied by breastmilk, they may not be present in sufficient quantity in infant formula milk. Milk fat globule membrane (MFGM) is a rich source of phospholipids, including sphingomyelin and has been associated with improved cognitive development in infants and children when added to infant formula. To determine if added bovine MFGM is also associated with improved myelination, this study used myelin-sensitive MRI to compare myelination trends in healthy infants and toddlers, 0–2 years of age, who received the same branded infant formula with and without added bovine MFGM in two temporal cohorts: *Without Added MFGM* between 2010 and 2017; and *With Added MFGM* between 2018–2020. Concurrent with imaging, cognitive development was assessed using the Mullen Scales of Early Learning (MSEL). Matched for important demographic and socioeconomic characteristics, we found that children who received infant formula with added MFGM showed improved myelination in motor-related areas (motor cortices, internal capsule, and cerebellum) and improved MSEL gross and fine motor scores. No significant differences in verbal or overall cognitive ability scores were noted. These results support the importance of phospholipids, sphingolipids, and sphingomyelin in promoting brain myelination and cognitive development.

## Introduction

The first 1000 days of a child’s life is a key period of neurodevelopment during which nearly all cognitive and behavioral abilities, or their precursory skills, begin to emerge (Johnson 2000). Underlying and supporting this rapid cognitive and behavioral development are equally profound changes in brain structure, microarchitecture, and functional connectivity (Mukherjee et al. 2002; Miller et al. 2003; Yoshida et al. 2013; Lebel and Deoni 2018; Edde et al. 2021), including the maturation of the cortical cytoarchitecture (via synaptogenesis, dendritic abhorization, and synaptic pruning), the advancement of the myelinated white matter, and a general shift in functional connectivity from localized intrahemispheric networks to longer-range interhemispheric ones (Edde et al. 2021). Myelination, which describes the wrapping of neuronal axons with the lipid myelin bilayer by oligodendrocytes, plays a key role in facilitating rapid and efficient brain messaging and connectivity by increasing the conduction velocity of electrical signals along the axon whilst also reducing the metabolic cost of signal transduction. The spatiotemporal pattern of myelination, often described as center-out and posterior-to-anterior, has been well described both histologically and, more recently, using non-invasive neuroimaging (i.e., magnetic resonance imaging, MRI) (Brody, et al. 1987; Deoni et al. 2012; Dean et al. 2014a), and mirrors the emergence of cognitive skills (Fornari et al. 2007; Fields 2008, 2015; Chevalier et al. 2015).

The myelination process is driven by genetic and environmental factors, with the oligodendrocytes stimulated by electrical activity along the axon to form the myelin sheath on individual and neighboring axons (Demerens et al. 1996). This stimulation, however, must be paired with adequate delivery of key compositional building blocks, which are often provided by nutritional intake. The myelin sheath is uniquely high in lipid content. It contains the bulk of the brain’s lipid content, with all lipid classes contained within its structure, including cholesterol (25–28% of total lipid weight), galactolipids (27–30%), and phospholipids (40–45%) (Kister and Kister 2022). Nutritional deficiencies, including iron (and transferrin), choline, and long-chain polyunsaturated fatty acids (LC-PUFAs), prior to and during early neurodevelopment can severely alter myelination, reduce levels of insulin-like growth factors (critical to oligodendrocyte development), and limit up-regulation of myelin protein genes (Connor and Menzies 1996; Todorich et al. 2009; Skripuletz et al. 2015; Siegert et al. 2017).

Provided adequate nutritional status, a mother’s own milk is recognized as the standard for providing the nutritional needs in early childhood (i.e., the first 3–6 months of infancy), including those nutrients needed for neurodevelopment. However, when breastfeeding is not possible, not chosen, or insufficient (i.e., inadequate infant growth and failure to thrive), infant formula must be depended upon. Over the past several decades, the nutritional composition of formula milk has improved significantly through the addition of LC-PUFAs such as docosahexaenoic and arachidonic acids (DHA and ARA, respectively), iron, prebiotics such as human milk oligosaccharides, and other nutritional factors intended to more closely mimic breastmilk. Recently, increased interest has been paid to the role of added gangliosides and phospholipids in infant formulas, such as through the addition of milk fat globule membrane (MFGM) (Silva et al. 2021). MFGM refers to the phospholipid trilayer that encases the milk fat globule and though it only accounts for 4% of the volume of the milk fat globule it contains the majority of milk gangliosides, phospholipids, sphingolipids, and sphingomyelin—critical components of the myelin sheath.

Past observational and randomized clinical trials in preterm and full-term infants, as well as in older children and adolescents, have suggested improved neurodevelopment in children who received infant formula with added bovine MFGM (Timby et al. 2014; Li, et al. 2019; Ambrozej, et al. 2021). These cognitive improvements include early cognitive and motor development, socioemotional skills, processing speed, and executive functioning (i.e., working memory) abilities. Given the role and importance of phospholipids in the composition of the myelin sheath, it may be hypothesized that observed neurodevelopmental outcomes may be the result of increased myelination.

Magnetic resonance imaging (MRI) provides a noninvasive method of quantifying myelin content and myelination throughout infancy and across the lifespan. Past work has further shown the ability of myelin-sensitive MRI to quantify myelination change associated with breastfeeding and nutritional intake, which may provide insight into the impact of added MFGM intake on infant myelination and neurodevelopment.

The Brown university Assessment of Myelination and neuroBehavior Across Maturation (BAMBAM) cohort offers a unique opportunity to examine potential differences in myelination patterns in children associated with infant nutrition intake via natural history experiments. BAMBAM is an accelerated longitudinal cohort study (Deoni et al. 2012; Dean et al. 2014a) aimed at characterizing neurodevelopment from infancy through adolescence in a large community sample of healthy and neurotypically developing children since 2009. Now part of the NIH Environmental influences on Child Health Outcomes (ECHO) program (Blaisdell et al. 2022), the BAMBAM sample includes approximately 1300 healthy children who were recruited between 0 and 24 months of age and have received repeated MRI scans and neurocognitive assessments, as well as routine biological samples, nutritional intake diaries, and home, environmental, medical, and educational updates. In general, children in BAMBAM follow a biannual visit schedule from 0–2 years of age and yearly thereafter.

Neuroimaging includes assessments of gross brain growth and morphometry via anatomical imaging, myelin content via multicomponent relaxometry, tissue microstructure via diffusion tensor imaging, and functional development using resting-state functional connectivity. In children 0 to 68 months of age, the Mullen Scales of Early Leaning, MSEL (E.M., M., Mullen Scales of Early Learning. 1995), are used to assess cognitive and motor abilities.

Nutritional intake information in infants is collected via parental questionnaires that ask about breastfeeding or formula feeding practices (including the ratio of feeding methods, duration of exclusive breastfeeding, type and brand of formula used). In this study, this parent-provided information was used to identify children who exclusively received infant formula, the brand, and whether the formula contained additional added bovine MFGM.

With respect to early infant feeding and nutrition, in early 2018 a major US formula manufacturer (Mead Johnson Nutrition) introduced a commercially available whey protein concentrate (source of added bovine MFGM) to a routine cow’s milk-based infant formula (Enfamil NeuroPro).

Based on a child’s enrollment into the BAMBAM cohort we identified two temporally separate cohorts of children between 0 and 2 years of age and who exclusively received the same commercially available infant formula with and without added bovine MFGM. *Without added MFGM:* n = 43 children who were recruited and followed from 0 to 2 years of age from January 2010 to December 2017; *With added MFGM:* n = 28 who were recruited and followed from April 2018 to March 2020.

Using retrospective data from these two child cohorts, matched for age, male: female ratio, maternal education, and other socioeconomic and demographic indicators, we compared myelination patterns throughout the brain and cognitive measures assessed using the Mullen Scales of Early Learning. We limited our analyses to the first two years of life due to the timing of the COVID-19 pandemic (January-March 2020), which profoundly disrupted the early child home, daycare, and education environments.

## Methods

All data presented in this work were collected in accordance with ethical approval and oversight by the Rhode Island Hospital Institutional Review Board. Informed consent was obtained from all parents and/or legal guardians.

### Study design and participants

**Fig. 1:**
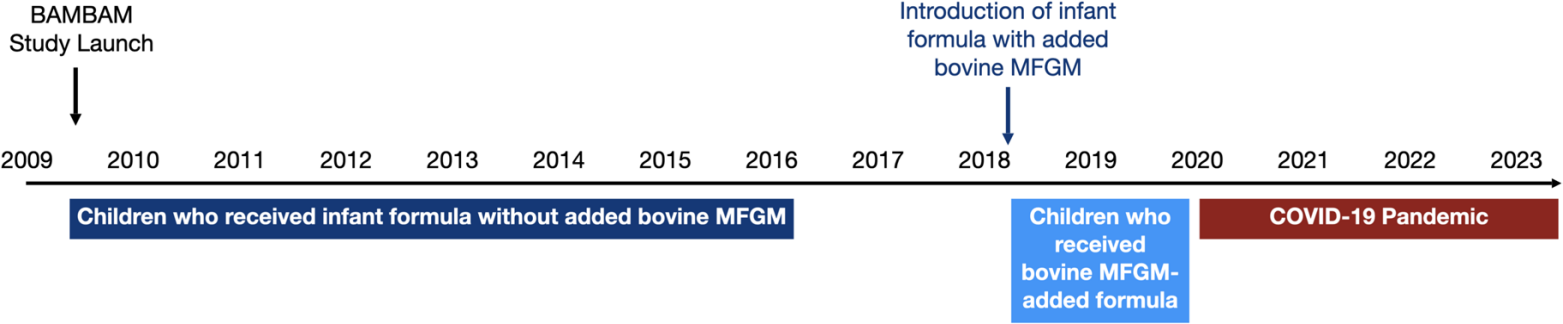
A timeline of the BAMBAM study shows study initiation, the introduction of the MFGM-added formula, the onset of the COVID-19 pandemic, and the windows during which children for this analysis were born

**Fig. 2:**
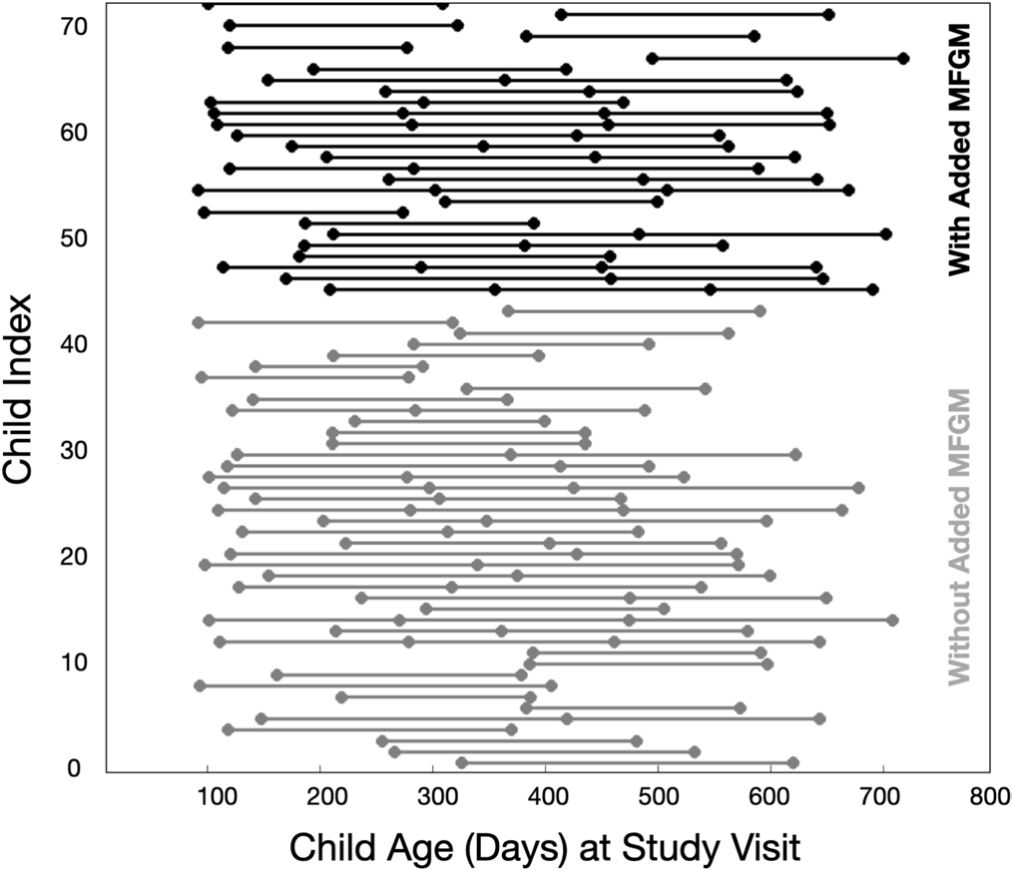
Overview of the collected longitudinal data used in the current analysis. Each line represents a single child, with dots denoting a study visit. All children had at least 2 study visits with high quality MRI and MSEL data collection, and parent reported feeding information

A temporal timeline of the BAMBAM study is shown in Fig. 1. Initiated in mid-2009, the study has continuously enrolled a total of 1307 healthy infants and toddlers from 2009 to the present. For this analysis, 43 (18 females) and 28 (10 females) healthy children were identified that met inclusion/exclusion criteria (described below), from the BAMBAM cohort who received the same commercially available infant formula with and without added bovine MFGM, respectively. These children provided a total of 107 and 78 longitudinal neuroimaging and neurocognitive datasets, respectively (illustrated in Fig. 2.)

**Fig. 3:**
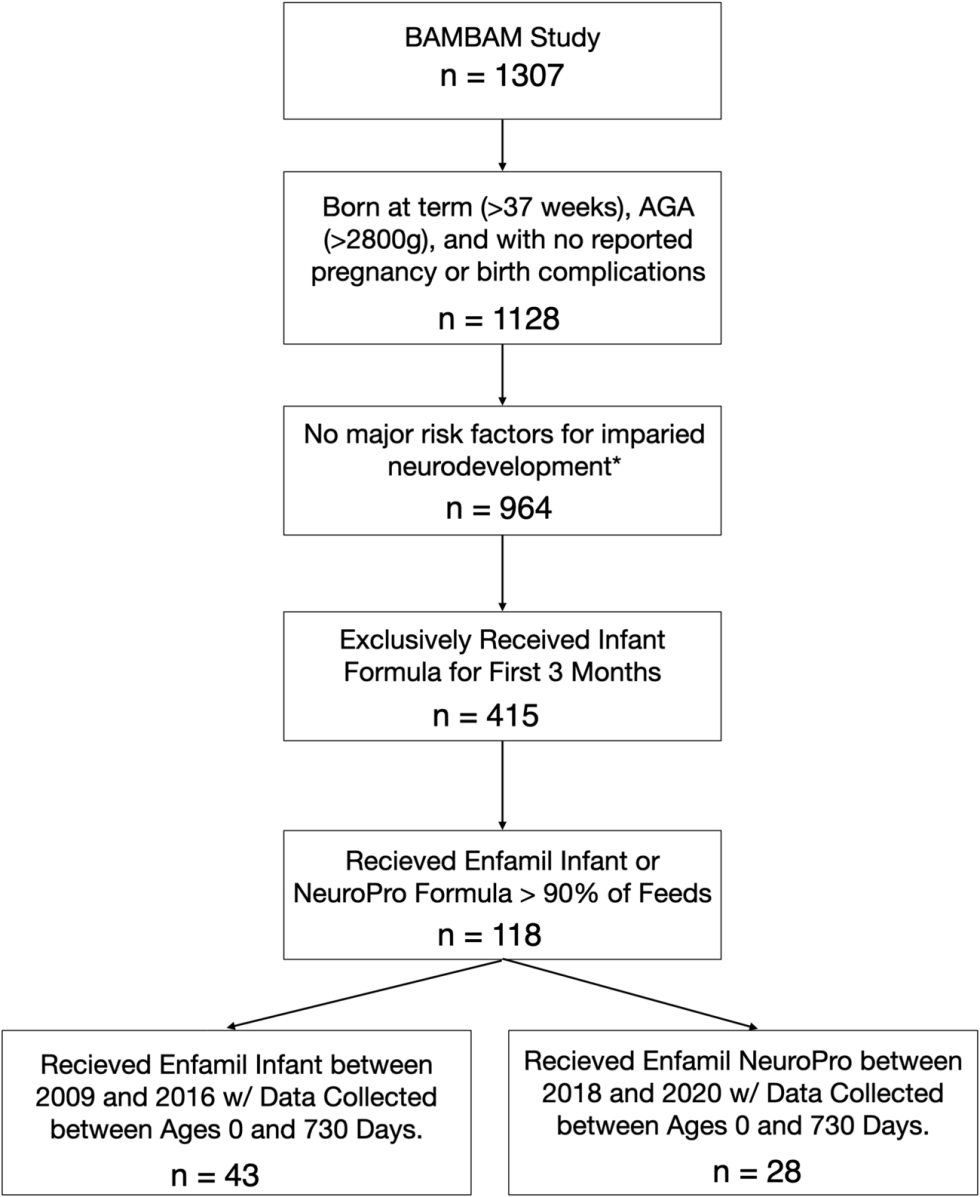
Flow chart showing how are final sample of 71 children was selected from the larger BAMBAM study cohort. *Risk factors for impaired neurodevelopment included preterm birth or low birthweight, complications during delivery that required a NICU stay, in utero exposure to cigarettes, alcohol, or illicit substances, a family history of learning or psychiatric disorders (including major depressive disorder in the mother requiring medication during pregnancy), and history neurological trauma or disorder

A flow chart showing how we arrived at the two child cohorts is shown in Fig. 3 based on the inclusion/exclusion criteria described below. All children were exclusively formula-fed, as defined by parent-report of at least 90% of feeds from infant formula over the first three months of life. Children in the standard infant formula (Enfamil Infant, without added bovine MFGM) group were born between June 2009 and December 2017 and followed to December, 2018. Children in the added bovine MFGM infant formula group (Enfamil NeuroPro) were born after April, 2018 and followed to March, 2020. All parents provided information on feeding practices, including the number and quantity of feeds per day (formula and breastfeeding) and infant formula brand(s) consumed.

We limited our analysis to the first two years of life to avoid the confounding effects of the COVID-19 pandemic. The pandemic brought significant public health measures (daycare and preschool closures, work-from-home and shelter-in-place orders) that disrupted the usual home and child development environment and likely affected early child development.

To focus on neurotypical development, we excluded children who: 1. Were born pre-term (less than 37 weeks); 2. Small for gestational age (< 2800 g); 3. Were non-singleton pregnancy; 4. Had a complicated delivery that required a stay in the NICU or 5-min APGAR scores less than 8; 5. Had in utero exposure to cigarettes, alcohol, or illicit substances; 6. Had a family history of learning or psychiatric disorders (including major depressive disorder in the mother requiring medication during pregnancy); and 7. Have experienced neurological trauma or disorder (e.g., birth asphyxia, epilepsy). Demographic details of each infant formula group are provided in Table 1.

**Table 1:** Demographic overview of the included child cohort. Mean values are provided with, where appropriate, standard deviations in brackets. *p* Values were calculated using a two-tailed student’s unpaired t-test and chi square test (for child sex and marital status)

		Without Added MFGM	With Added MFGM	p Value
Child Sex	Male (n)	25	18	0.6
	Female (n)	18	10	
# Scans per Child		3 (1.2)	2.8 (1.7)	0.56
Age Range (days)		98—708	92—717	
Mean Gestation (days)		275 (11)	276 (7)	0.67
Mean Birth Weight (g)		3179 (555)	3313 (540)	0.32
Mean Birth Length (inches)		20 (1.8)	21 (3.5)	0.12
Mean Maternal Education (HHS)		5.4 (1.1)	5.6 (1.5)	0.52
Mean Inter-Scan Period (days)		307 ± 199	330 (190)	0.60
Mean # of Months of Exclusive Formula Intake		6.15 (2.06)	5.77 (1.89)	0.43
Marital Status	Married / Living Together (n)	34	22	0.96
	Divorced / Single (n)	9	6	

There were no significant group differences in various birth and socioeconomic characteristics, including gestation duration, mean birth weight and length, maternal education (codified using the Hollingshead Scale (Cirino et al. 2002)), and marital status (Table 1). All children were from homes where English was the first spoken language.

### Differences between formula compositions

Table 2 provides an abridged overview of the nutritional composition of the two investigated infant formulas, Enfamil Infant (without added MFGM) and Enfamil NeuroPro (with added MFGM). We have focused on nutrients previously shown and believed to be important to early infant brain development.

**Table 2:** Nutritional composition information for the two infant formulas examined in this study, highlighting nutrients believed to be important for early brain development

	Enfamil Infant	Enfamil NeuroPro
MFGM ingredient	–	^a^
Choline (mg/100 kcal)	24	24
Inositol (mg/100 kcal)	6	24
Iron (mg/100 kcal)	1.8	1.8
Folic Acid (mcg/100 kcal)	16	16
DHA (mg/100 kcal)	17	17
ARA (mg/100 kcal)	34	25

^a^ 6 g/L Lacprodan® MFGM-10, Arla Foods Ingredients, Basking Ridge, New Jersey

### MR imaging

Neuroimaging was performed on a Siemens 3 T Trio with a 12-channel head RF array. Children were typically imaged during natural, non-sedated sleep (Dean et al. 2014b). To minimize intra-scan motion, children were swaddled with an infant or pediatric MedVac vacuum immobilization bag (CFI Medical Solutions, USA), and foam cushions were packed around their heads. Scanner noise was reduced by derating the peak gradient amplitudes and slew rates to 25 mT/m/s. A noise-insulating insert (Quiet Barrier HD Composite, UltraBarrier, USA) was also fitted to the inside of the scanner bore. MiniMuff pediatric ear protectors and electrodynamic headphones (MR Confon, Germany) were also used for all children. A pediatric pulse-oximetry system and infrared camera were used to monitor children during scanning, and parents were allowed to sit with their child in the scanner suite, provided they had no MRI counter-indications.

To assess myelination, the mcDESPOT multicomponent relaxometry method (Deoni et al. 2008) was used to estimate voxel-wise measures of the myelin water fraction (MWF), a quantitative and surrogate measure of myelin content (MacKay and Laule 2016). Multicomponent relaxometry exploits the inherent sensitivity of T_1_ and T_2_ relaxivity to biochemical structure and composition to resolve sub-voxel water species. In the human brain, two such water species are consistently observed: a slow-relaxing water pool attributed to the intra/inter-cellular water and a fast-relaxing water pool attributed to water trapped within the lipid bilayers of the myelin sheath. The ratio of the myelin water to the non-myelin water pools (termed the MWF) is a non-invasive quantitative estimate of myelin content (MacKay and Laule 2016).

### Neurocognitive assessment

Within one week of a successful MRI, each child’s neurodevelopment was assessed using the Mullen Scales of Early Learning (MSEL (E.M., M., Mullen Scales of Early Learning. 1995)), a population-normed tool consisting of 144 items equally distributed across five main sub-domain: visual reception (VR), expressive and receptive language, EL and RL), and fine and gross motor function (FM and GM) in children 0 to 68 months of age. Each sub-test provides raw scores and age-normalized T-scores for each of the five sub-domains, which can be further combined into age-normalized composite scores reflecting overall ability, the Early Learning Composite (ELC), verbal, and non-verbal functioning (Verbal Development Quotient, VDQ, and Non-Verbal Development Quotient, NVDQ). ELC is derived from the fine motor, visual reception, and both language scores; VDQ from the two language scores, and NVDQ from the fine motor and visual reception scores. Scores for gross motor typically reach a ceiling by age 2 and are excluded from the composite measures.

### MRI and neurocognitive measure analysis

Following image acquisition, the mcDESPOT multicomponent relaxometry data were visually assessed for motion artifacts (e.g., blurring and ghosting) by the same researcher (SCLD) and MWF myelin content measures estimated throughout the brain on a voxel-wise basis using a 3-pool tissue model (Deoni et al. 2013). The quantitative MWF images were non-linearly aligned to MNI space using a multi-step, multi-scale approach as previously described (Deoni et al. 2012). The aligned images were then smoothed with a modest Gaussian kernel (4 mm full-width-at-half-maximum), and voxel-wise regression analysis was then performed using the following general linear model.

1 $$MWF_{i,j} = \beta_{0,j} + \beta_{1,j} log\left( {age_{i,j} } \right) + \beta_{2,j} biologialSex + \beta_{3,j} Group_{j} + + \beta_{4,j} log\left( {age_{i,j} } \right) \times Group_{j}$$

(Johnson 2000).

where.

*MWF*_*i,j*_ is the mean myelin water fraction estimate for child *j* at time-point *i*, *age*_*i,j*_ is the corresponding child age, Group_j_ is the child’s formula group (0 = Standard, 1 = Added bovine MFGM), *β*_*0,j*_ is the intercept and *β*_*1,j*_*, β*_*2,j*_* and β*_*3,j*_ are the regression coefficients that combine a sample fixed effect and a subject-specific random effect (u_j_), e.g.,

1a $$\beta_{0,j} = \beta_{o} + \mu_{0,j}$$

Voxels for which the Group x Age term was significant with an uncorrected *p*-value < 0.001 were then merged into a single region of interest (ROI). Mean MWF values for each child throughout the ROI were then calculated, and the regression analysis was repeated for this single ROI data.

Linear mixed-effects regression was also performed for the five MSEL sub-domain and three composite measures, i.e.,

2a $$VR_{i,j} = \beta_{0,j} Group + \beta_{1,j} age_{i,j}$$

2b $$FM_{i,j} = \beta_{0,j} Group + \beta_{1,j} age_{i,j}$$

2c $$GM_{i,j} = \beta_{0,j} Group + \beta_{1,j} age_{i,j}$$

2d $$EL_{i,j} = \beta_{0,j} Group + \beta_{1,j} age_{i,j}$$

2e $$RL_{i,j} = \beta_{0,j} Group + \beta_{1,j} age_{i,j}$$

2f $$ELC_{i,j} = \beta_{0,j} Group + \beta_{1,j} age_{i,j}$$

2g $$VDQ_{i,j} = \beta_{0,j} Group + \beta_{1,j} age_{i,j}$$

and,

2h $$NVDQ_{i,j} = \beta_{0,j} Group + \beta_{1,j} age_{i,j}$$

## Results

**Fig. 4:**
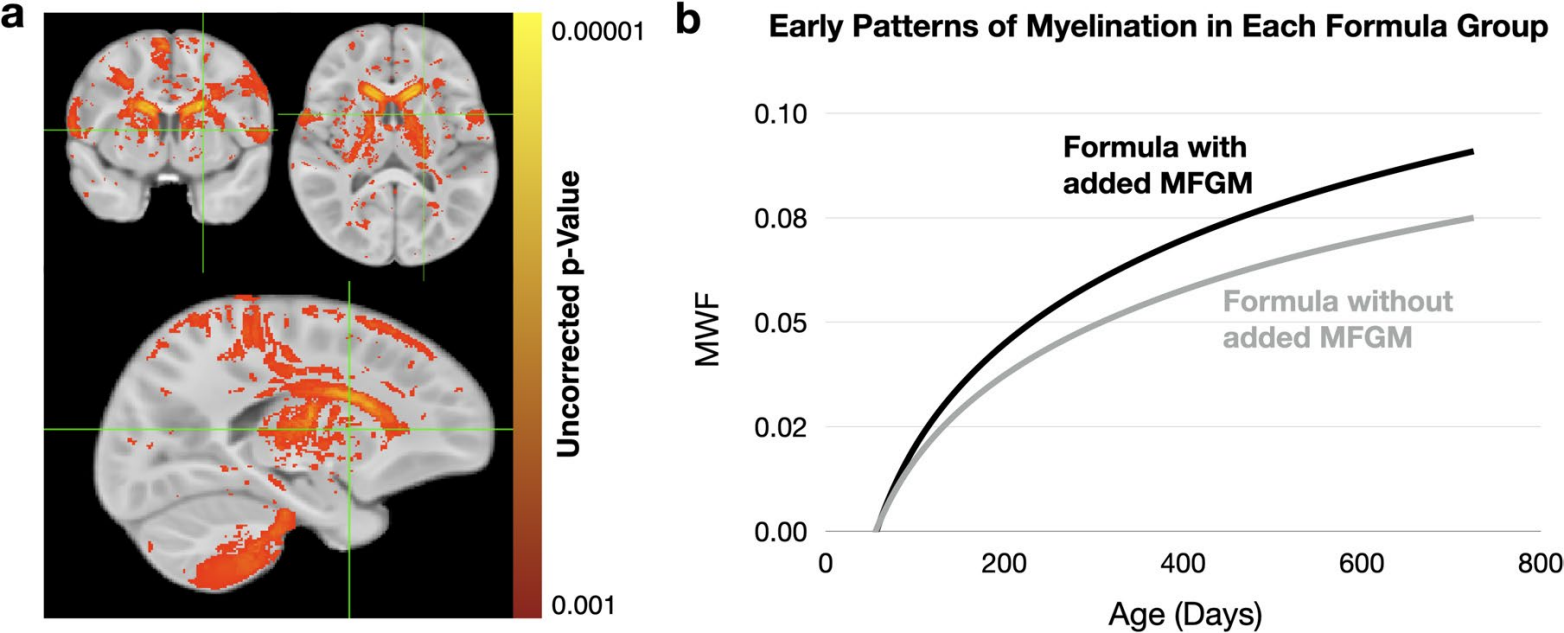
**a** Brain regions with a significant (*p* < 0.001 uncorrected) difference in myelination rate between the standard and bovine MFGM-added formula groups. **b** Modeled mean myelination trajectories for the ROI defined in *(a)* for each formula group, showing an overall increased rate of development and asymptotic value in the children who received the bovine MFGM-added formula

Figure 4a highlights brain areas where our voxel-wise regression analysis found an increased (*p* < 0.001 uncorrected) myelin development rate in the children who received the added bovine MFGM infant formula versus those who received the standard formula. These regions include bilateral portions of the cerebellum, primary and secondary motor cortices (and adjacent white matter), the caudate, thalamus, and internal capsule—all regions involved in movement planning, motor control, and sensory processing.

Using these highlighted voxels as a region of interest, mean myelination trajectories were modeled (Fig. 4b) using the same statistical methods described above. A statistically significant (*p* < 0.05) increase in myelination rate was found between the added bovine MFGM and standard infant formulas (Table 3), with an overall increase in myelin content of ~ 12% at age 2.

**Table 3:** Results of the mixed-effects regression analysis (described by Equation (Johnson 2000)) between the standard and bovine MFGM-added data. Overall, we note a significant increase in the MWF development rate (MFGM x Age) (*p* < 0.05) in the children who received the MFGM-added formula

Variable	Estimate	tStatistic	pValue
Intercept	−0.159518	−8.257426	< 0.001
Sex (Male)	0.000978	0.334574	0.738790
MFGM	**0.43809**	**1.987786**	**0.050132**
Age	0.35933	11.899496	< 0.001
MFGM X Age	**0.007557**	**2.161722**	**0.033519**

Bold entries denote a statistically significant result (*p* < 0.05)

**Fig. 5:**
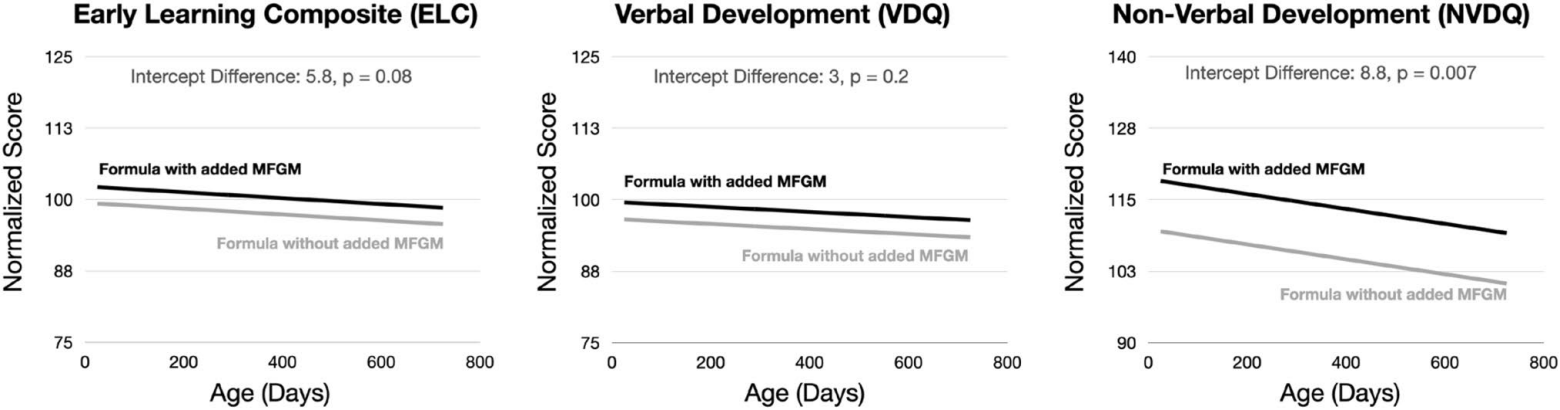
Mean longitudinal trends modeled for the three composite scores of the Mullen Scales of Early Learning. From the mixed-effects regression analysis (Table 3) we find a significant (*p* < 0.05) increase in NVDQ (motor and visual processing) in the children who received the MFGM-added formula

Examining the longitudinal cognitive data (Fig. 5), we find a significant (*p* < 0.006, corrected for multiple comparisons) increase in both the MSEL fine and gross motor sub-scores non-verbal functioning (Table 4) in the children who received the formula with added bovine MFGM, but no significant difference in language skills (EL or RL) or visual reception (VR). These differences further translate to an overall significant increase in the non-verbal processing composite (NVDQ), but not in verbal processing (VDQ) or overall cognition (ELC).

**Table 4:** Results of the mixed-effects regression analysis for each MSEL sub-domain and composite score. For the individual sub-domain scores, we find a significant group difference in gross and fine motor skills, which translates to an increased overall nonverbal development quotient (NVDQ), but no significant differences in other sub-domain or composite scores. These findings remain significant even after correcting for the multiple comparisons)

INDIVIDUAL DOMAIN SCORES					COMPOSITE SCORES				
	Variable	Estimate	t-Statistic	pValue		Variable	Estimate	t-Statistic	pValue
GM	Intercept	52.5269	29.6888	< 0.001	ELC	Intercept	102.2996	30.3888	< 0.001
	Age	− 0.0018	− 1.7147	0.0876		Age	− 0.0052	− 1.7570	0.0819
	Group	− 7.1623	− 3.6212	0.0010		Group	− 5.7710	− 1.7668	0.0802
FM	Intercept	54.6474	24.1512	< 0.001	VDQ	Intercept	99.6436	22.0781	< 0.001
	Age	− 0.0048	− 2.4222	0.0172		Age	− 0.0044	− 1.1284	0.2618
	Group	− 6.7932	− 3.0942	0.0030		Group	− 2.9920	− 0.6833	0.4960
VR	Intercept	54.6680	22.7010	< 0.001	NVDQ	Intercept	118.6030	35.3889	< 0.001
	Age	− 0.0035	− 1.6491	0.1022		Age	− 0.0130	− 4.4592	< 0.001
	Group	− 3.1672	− 1.3555	0.1783		Group	− 8.8080	− 2.7087	0.0080
EL	Intercept	47.7722	18.7735	< 0.001					
	Age	− 0.0005	− 0.2453	0.8067					
	Group	− 1.5745	− 0.6377	0.5251					
RL	Intercept	48.1815	23.0386	< 0.001					
	Age	− 0.0026	− 1.4074	0.1623					
	Group	− 1.6335	− 0.8050	0.4227					

## Discussion

Nutrition has long been considered a potentially modifiable variable affecting child health and neurodevelopment. In this preliminary study, we sought to examine the impact of added bovine MFGM, a rich source of phospholipids, sphingolipids, gangliosides, cholesterol, and proteins (Lee et al. 2018) on infant neurodevelopment. Past in vitro and in vivo research has shown that sphingolipids, specifically sphingomyelin, are important compositional components of the myelin sheath (Poitelon et al. 2020) that help maintain myelin structure and support various signal transduction pathways (Hannun 1994). In an in vitro model of neurodevelopment (Sprague Dawley rat pups), increased sphingomyelin intake has been associated with increased proliferation, maturation, and differentiation of oligodendrocyte precursor cells (OPCs), as well as increased axon myelination (Schneider, et al. 2019). These results have also been extended into early childhood (Schneider, et al. 2019, 2023), with observational and randomized control trials showing improved neurodevelopmental outcomes in infants who have received infant formula supplemented with additional bovine MFGM (Timby et al. 2014; Li, et al. 2019; Ambrozej, et al. 2021).

The present work builds on and extends this past evidence, with novel neuroimaging data collected in children who reviewed either a standard commercially available infant formula or a more recently introduced formula with added bovine MFGM, suggesting increased intake of bovine MFGM is associated with improved myelination in various early-maturing brain regions, in particular those involved in motor and sensory processing. Mirroring these neuroimaging findings, assessments of fine and gross motor control show improvements in children who received the added bovine MFGM. These neuroanatomical findings are also supported by existing literature showing increased processing speed—the primary role of myelin in the brain—in children at 5.5 years of age who received supplemented with MFGM (Colombo, et al. 2023). Cumulatively, these results support the role of sphingomyelin (alone or in combination with the other phospholipids, sphingolipids, and gangliosides in MFGM) in improving early infant neurodevelopment (Hernell et al. 2016).

However, it is important to note three potential caveats in this work. First, unlike past clinical trials (Schneider, et al. 2023), this study was not a randomized controlled trial. Rather, our results are derived from retrospective data collected from children enrolled in an ongoing longitudinal study of healthy brain development. Children were drawn from two temporal epochs corresponding to the market availability of similar cow's milk-based formulas with and without added bovine MFGM. As this was not a rigorous clinical trial, there were no controls or measurements of the amount of feeding and, therefore, the amount of MFGM consumed by each child is unknown. Further, increased inositol was also added alongside the bovine MFGM (Table 2), which also plays an important role in early neurodevelopment, specifically for synaptogenesis and functional connectivity (Paquette et al. 2023). As neural activity partly drives myelination (Gibson et al. 2014), inositol may also indirectly promote myelination through increased synaptic activity and function. We are unable, therefore, to ascribe our findings solely to MFGM, inositol, or their combination.

Second, the sample size was relatively small, with approximately 40 and 30 children in each infant formula group. Based on past estimates of myelin imaging variability across the investigated age range (Deoni et al. 2012), this sample size is sensitive only to moderate-to-large effects (7–8% difference in myelin content). Examining the myelin modeling results (Table 2), the Cohens D for the MFGM x Age term was 0.53, representing a medium effect size.

Finally, data was only analyzed over the first two years of life. It is possible that additional effects could have been found if the age range had been extended to 4 or 5 years. For example, additional cognitive skills such as executive functioning). However, we were limited by the impact of the COVID-19 pandemic and related changes in each child and family’s home, school, and work environments. Emerging work has suggested that cognitive development was affected throughout the earliest stages of the pandemic (Firestein et al. 2023), and it is unclear how to adequately control for those effects.

Despite these caveats, results presented in this work offer tantalizing supporting evidence that improved nutrition throughout infancy, in particular through added bovine MFGM intake, may significantly improve early brain myelination and neurodevelopment.

## Conclusion

The importance of early infant nutrition in child health and neurodevelopment has been increasingly recognized through studies examining iron, folic acid, vitamins A, B12, C, D, and K, choline, DHA, and other macro and micronutrients. In this work, additional evidence supports other emerging literature reports espousing the neurodevelopmental benefits of MFGM intake in infancy. Specifically, results demonstrate improved myelination associated with increased neurocognitive performance in children who received infant formula with added bovine MFGM. Further work within a more rigorous randomized control framework is needed to confirm these findings.

## Data Availability

All data used for the included analysis are available upon request.

## Abbreviations

ARA: Arachidonic acid
DHA: Docosahexaenoic acid
ELC: Early Learning Composite score from the Mullen Scales of Early Learning
LC-PUFA: Long-chain polyunsaturated fatty acid
MFGM: Milk Fat Globule Membrane
MRI: Magnetic Resonance Imaging
MSEL: Mullen Scales of Early Learning
MWF: Myelin Water Fraction
NVDQ: Non-Verbal Development Quotient score from the MSEL
VDQ: Verbal Development Quotient score from the MSEL
